# Effects of Steam Heat and Dry Heat Sterilization Processes on 3D Printed Commercial Polymers Printed by Fused Deposition Modeling

**DOI:** 10.3390/polym14050855

**Published:** 2022-02-22

**Authors:** Jorge Mauricio Fuentes, Marina Patricia Arrieta, Teodomiro Boronat, Santiago Ferrándiz

**Affiliations:** 1 Departamento de Ingeniería Mecánica y de Materiales, Instituto de Tecnología de Materiales, Universitat Politécnica de València, Plaza Ferrándiz y Carbonell s/n, 03801 Alcoi, Spain; tboronat@dimm.upv.es; 2 Ingeniería en Diseño Industrial, Facultad de Ingeniería y Ciencias Aplicadas, Universidad Central del Ecuador, Quito 170521, Ecuador; 3 Departamento Ingeniería Química Industrial y Medio Ambiente, Universidad Politécnica de Madrid, E.T.S.I. Industriales, 28006 Madrid, Spain; 4 Grupo de Investigación: Polímeros, Caracterización y Aplicaciones (POLCA), 28006 Madrid, Spain

**Keywords:** fused deposition modeling, 3-D printing, single-use surgical devices, moist heat sterilization process, dry heat sterilization, PLA, PETG, CPE

## Abstract

Fused deposition modeling (FDM), the most widely used additive manufacturing (AM) technology, is gaining considerable interest in the surgical sector for the production of single-use surgical devices that can be tailor-made according to specific requirements (e.g., type of patient surgery, specific shapes, etc.) due to its low cost, ease of access to materials (3D-printing filament), and the relatively low complexity. However, surgical 3D-printing parts should resist sterilization treatments without losing structural, mechanical, and dimensional accuracy. Thus, in this work, 3D-filaments based on poly(lactic acid) (PLA), poly(ethylene glycol-co-1,4-cyclohexanedimethanol terephthalate) (PETG), and a modified PETG material (CPE) were used to produce 3D-printed parts and further subjected to moist heat (MH) and dry heat (DH) sterilization processes as affordable and widely used sterilization processes in the medical field. The effect of MH and DH was evaluated by performing a complete mechanical, structural, thermal, and morphological characterization before and after both treatments. In general, the moist heat treatment produced a higher degradation of the polymeric matrix of PETG and CPE due to hydrolytic and thermal degradation, particularly affecting the tensile test and flexural properties. For instance, the linear coefficient of thermal expansion (LCTE) before glass transition temperature (T_g_) increased 47% and 31% in PETG samples due to the MH and DH, respectively, while it increased 31% in CPE due to MH and was mainly maintained after the DH process. Nevertheless, in PLA, the MH produced an increase of 20% in LCTE value and the DH showed an increase of 33%. Dry heat treatment resulted in being more suitable for medical applications in which dimensional accuracy is not a key factor and there are no great mechanical demands (e.g., surgical guides).

## 1. Introduction

During surgical planning, specific surgical devices are required and the commercially available surgical devices do not always fit the specific conditions required to manipulate the external devices. For instance, many surgical devices need to work within multiple quadrants without repositioning the surgical parts [1]. Additionally, many single-use surgical devices (e.g., screws) need a specific design to be used only during the surgery for a patient to properly introduce prosthesis into the human body but are disposed of after surgery. Thus, additive manufacturing (AM), colloquially known as 3D printing, has gained considerable interest in this field, mainly because of its ability to easily produce made-to-order products at low cost [2]. In this context, over the most typical plastic processing technologies (e.g., extrusion, injection molding, etc.), AM permits the fabrication of fully customized products with geometrical complex structures in a simple, fast, and economic manner [3]. Several polymers (polylactic acid (PLA) [4], poly-ε-caprolactone (PCL) [5], etc.) can be processed into 3D printing materials and are further used for medical applications such as tissue engineering, wound handling, drug supply orthopedic devices, surgical devices, and operating guides [6,7,8]. Nevertheless, the promise of 3D printing is based on custom products that are made to order in unique configurations (e.g., dental and/or medical devices, and low-turnover replacements parts) [2]. Among the other AM technologies, the fused deposition modeling (FDM) process is the most commonly used by prototyping and product manufacturers [9]. FDM is based on the layer-by-layer deposition of a thermoplastic polymer at temperatures above its melting temperature (T_m_), thus the material changes from a solid to a semi-liquid state during the extrusion process and follows the path of a computer-aided system [10]. The 3D-filament roll passes through a heated nozzle of extrusion that deposits in coordinates in x and y coordinates, while the print surface lowers the object layer by layer in the Z direction. In this way, the object is printed from the bottom-up method. If a model has protruding parts, support structures will be needed that can be removed once the 3D-printing process is completed [7].

AM is currently available in many hospitals for the development of personalized medical devices and implants [11] and several filaments for 3D printing are commercially available as well including biocompatible polymers such as PLA. However, the materials for surgical applications should be sterilizable [8]. Among the sterilization processes used to properly remove microorganisms in medical devices such as chemical (e.g., ethylene oxide, hydrogen peroxide, etc.), mechanical (e.g., filtration), and physical (heat and radiation), the heat sterilization process is very interesting since it allows for the sterilization of virtually all organisms with no toxic residues through a simple process and is currently available in many hospitals [12,13]. Heat sterilization is typically divided into steam sterilization conducted by moist heat (MH) and in dry heat (DH) [13].

Poly(lactic acid) (PLA) is a material widely used in the biomedical sector since it is approved by the Food and Drug Administration (FDA) due to its biocompatibility and because its degradation products are non-toxic [4,10,14]. It is also widely used in 3D printing processes, mainly due to its low glass transition temperature (58 °C) and low melting point (175 °C) [11]. In particular, PLA-based 3D printing materials have massively matured during the worldwide COVID-19 pandemic, which has allowed for the mitigation of the shortage of critical supplies in the fight against the pandemic (e.g., personal protective equipment (PPEs), medical devices, and testing devices) in the framework of a circular economy approach [15,16,17]. PLA filament for 3D printing can be found in a wide variety of compositions. For instance, some trademarks use reinforcing fillers (e.g., ceramic particles and wood-derived or colorants that are ultimately compatible with most 3D printers [3,4]. For the 3D-printing process, PLA does not require a high temperature in the printing bed (less than 60 °C), while the working temperature should be set between 195 °C and 220 °C [4,18]. Today, PLA is one of the most widely used sustainable polymers in many applications due to its biobased origin, biodegradable character, and recyclability [16,19,20]. However, PLA has high strength and stiffness, resulting in a brittle material. Therefore, PLA’s low resistance to sterilization processes is expected to limit its wide use in single-use surgical devices. Frizziero et al. (2021) studied 3D printed parts based on PLA subjected to a regular heat-sterilization cycle and they observed that 3D-PLA-based parts mainly maintained the geometry after a moist heat (MH) sterilization process conducted in an autoclave [21]. However, although PLA can maintain its geometry after the sterilization process, the mechanical resistance of such surgical devices is of fundamental importance and should be extensively studied.

There are other commercial 3D printing filaments commercialized under the trade name CPE by Ultimaker (CPE-HG 100 Extrafill), which is an amorphous co-polyester characterized as being durable and tough. According to the supplier, this co-polyester contains a bio-based monomer and can be recycled. CPE has higher tensile flexural and impact strength than PLA and possesses higher thermal and chemical resistance than PLA filament. CPE filament has great optical properties, very high gloss, and clarity. It is used in kitchen instruments since it is bis-phenol A (BPA) free. Its recommended printing temperature is 260 °C, while the printing bed temperature ranges from 70 °C to 80 °C.

Another commercial filament is based on poly(ethylene glycol-co-1, 4-cyclohexanedimethanol terephthalate) (PETG), which is a co-polyester of terephthalic acid with ethylene glycol (≥50%) and cyclohexanedimethanol (CHDM) (≤50%). It is used to replace polyethylene terephthalate (PET) in certain applications where PET transparency is required because PET tends to crystallize, which decreases its transparency and severely limits its use. Thus, to avoid crystallization, a glycol-modified PET co-polymer has been copolymerized. The commercial filament is reinforced with carbon fibers (CF), resulting in polymeric formulations with mechanical properties of PETG that are close to that of PET. PETG+CF is an amorphous polymer when the CHDM content is in the range of 32–62% [22]. PETG’s typical uses are medical devices, food-contact applications, and personal care packaging. According to the supplier, the first layer temperature should be 240 °C and the other layer temperature a 250 °C, while the printing bed temperature should be around 90 °C.

Although 3D printing products have gained considerable interest during the last years, to the best of our knowledge, there are no studies where the effect of moist and dry heat sterilization processes on the structural and mechanical properties of PLA-based 3D printing parts has been studied. Thus, in this work, to extend 3D-printing products in the field of sustainable single-use surgical devices such as screws or guides that are only used in particular surgeries and with required designs for each patient, the effect of the heat sterilization processes, currently available in many hospitals, was evaluated. Therefore, the effect of dry heat as well as steam heat or moist heat on the sterilization processes of 3D-printed PLA processed through FDM was evaluated and compared with two typical commercial 3D filaments: CPE and PETG+CF. Thus, the three polymeric filaments were processed by FDM into 3D-printed products. The obtained materials were characterized before and after the dry heat (DH) and moist heat (MH) sterilization processes using the determination of the mechanical properties (tensile, flexural, impact, and hardness tests), thermal (thermogravimetric analysis (TGA), and differential scanning calorimetry (DSC). The chemical changes on specific functional groups were studied by Fourier transform infrared (FTIR) spectroscopy while the changes in the visual and structural appearance were followed by colorimetric measurements and stereomicroscopy observations.

## 2. Materials and Methods

### 2.1. Materials

Three types of commercial materials used for the test included 3D printed, PETG+CF supplied from Nanovia (T_g_ = 80 °C), CPE from Fillamentum (CPE-HG 100 Extrafill, T_g_ = 82 °C), and PLA from Prusament (T_g_ = 57 °C and Tm = 115 °C).

### 2.2. Samples Preparation and Sterilization Processes

The 3D printed samples were modeled using the Solidworks 2017 software into different test specimens (rectangular probes and dog-bone style samples for mechanical testing) and then saved as an STL file. Finally, to generate G code, a Slic3r Prusa Edition 1.41.i3 MK3 software was used. The materials were printed by means of an Original Prusa i3 MK3s 3D printer (Partyzánská, Praha, Czech Republic). Main printer parameters were preconfigured for each filament in Slic3r and can be seen in the Supplementary Materials (Table S1). The 3D printed materials were designed based on the polymeric material used, followed by the infill percentage (IP) in which the type of filling was identified with the prefix L or H for rectilinear and honeycomb, respectively. PETG+CF, CPE, and PLA filaments were printed with the 80% and 40% infill and rectilinear and honeycomb infill type of the Slicer Prusa Edition software, as these infill types are the most commonly used [23].

Two heat sterilization processes were evaluated: dry heat (DH) and moist heat (MH). Dry heat (DH) sterilization was conducted in a Selecta oven (Selecta S.A., Barcelona, Spain). The 3D-printed specimens were placed on trays and subjected to the following cycle of temperature: (1) heating up from room temperature to 140 °C in 70 min; (2) maintained at 140 °C for 3 h to perform the sterilization process itself; and (3) cooling from 140 °C to room temperature in approximately 4 h. For the moist heat (MH) sterilization process, a saturated water vapor ambient condition was generated in a pressure cooker Monix Classica (ISOGONA, SL, Valls, Tarragona, Spain). The specimens were placed on a metal support and subjected to a 45-min sterilization cycle consisting of (1) a heating cycle up to 121 °C in 5 min; (2) maintained at 121 °C during 20 min; and (3) cooled down from 121 °C to room temperature. The temperature was monitored with a Raytek RS373-8499 infrared thermometer (Raytek, Raynger St, Santa Cruz, CA, USA).

Table 1 summarizes the formulations printed in this study, the sterilization processes used in each sample as well as their labeling. For instance, the material PLA-L-80-MH is PLA with rectilinear printing at 80% fill percentage sterilized by a moist heat process.

**Figure 1:**
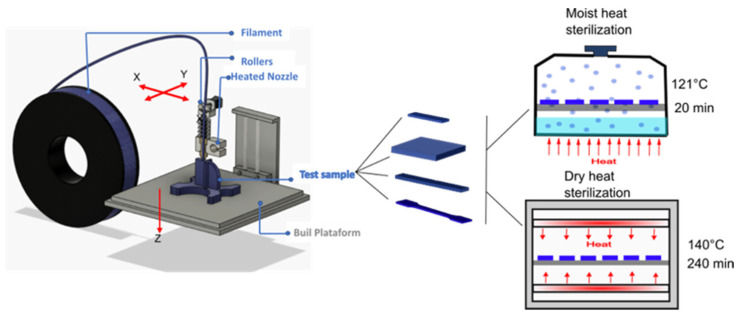
Schematic representation of the 3D-printing process followed by the sterilization processes.

Figure 1 shows a schematic representation of the 3D printing process, the 3D printed samples obtained, and the sterilization processes used in this work.

### 2.3. Samples Characterization

#### 2.3.1. Mechanical Characterization

##### Tensile and Flexural Tests

The tensile tests as well as flexural properties of 3D-printed samples before and after the sterilization processes were determined using a universal test machine (Ibertest Elib 30 of SAE Ibertest (Madrid, Spain)) at room temperature, according to ISO 527-1 [24] and ISO 178 [25], respectively. Dog-bone samples were prepared with a length of 75 mm, a width of 5 mm, and a thickness of about 2 mm. The tests were carried out with a 5 kN load cell and the speed of the tests was set at mm/min in both cases and at least five specimens were tested for each formulation.

##### Hardness

A Shore D hardness was measured according to ISO 868 [26], with a 676-D model hardness tester from J. Bot Instruments Vilassar de Dalt JBA (Barcelona, Spain). The Shore D hardness was measured on a 7 mm thick square and 50 mm of the side printed on the XY plane using the profile preconfigured printed for each material (see Table 1), but with 100% infill density and a layer thickness of 0.2 mm. The 3D file was generated in SolidWorks 2017 and saved as STL to print and generate the G code; the printer’s own Slic3r software was used. The specimens were adjusted to these dimensions so that the definitions in points 5 and 6 of ISO 868 were complied with, in order to perform five penetrations in each piece, while at least five specimens were tested for each formulation and the mean and standard deviation of each mechanical property are reported.

##### Charpy Impact Test

The resistance to impact tests was carried out according to ISO 179 [27] with a 1-J Charpy pendulum from Metrotec (San Sebastián, Spain). All tests were performed at room temperature, assaying at least five samples per test. All specimens were printed in the horizontal direction. The test specimens for the flexural test had the dimensions recommended in ISO 178, thus their dimensions were 10 × 4 × 80 mm^3^. Charpy impact test specimens were printed horizontally following the honeycomb and rectilinear fill patterns with 80% and 40% fill. Five samples of each configuration type were used for the impact test and the mean and the standard deviation were reported. The specimens for the CPE-based 3D-printed material were mechanically notched to produce their breakage due to their high flexibility; in all cases, a 1 KJ hammer was used. At least five specimens were tested for each formulation.

#### 2.3.2. Microstructural Analysis

The fracture surfaces from the samples assayed from the Charpy impact test were observed in an Olympus SZX7 (Tokyo, Japan) stereomicroscope with a KL-1500-LCD light source. At least five specimens were tested for each formulation.

#### 2.3.3. Thermomechanical-Analysis

The thermo-mechanical analysis was used to evaluate the changes in the dimensions of the material as a function of temperature by calculating the linear coefficient of thermal expansion (LCTE) according to ASTM E831-19 by means of Q400 TA TMA equipment from TA Instruments (New Castle, DE, USA). The test specimens’ dimensions were 3 × 3 × 3 mm^3^. The expansion method was used with a heating temperature from 23 °C up to 140 °C at 5 °C min^−^^1^ with a weight of 0.001 N. At least five specimens were tested for each formulation

#### 2.3.4. Colorimetric Measurements

The colorimetric properties in the CIELab space were measured with Konica CM-3600d Colorflex-DIFF2 equipment from Hunter Associates Laboratory, Inc. (Reston, VA, USA). The color coordinates L (lightness), a*(+a* = red, −a* = green), and b*(+b* = yellow, −b* = blue) were acquired. Measurements were performed five times on each sample and the mean value of at least five samples with the corresponding standard deviation was reported.

#### 2.3.5. Thermal Characterization

##### Thermogravimetric Analysis

The thermal stability of filaments, 3D-printed materials, and the sterilized 3D-printed samples was evaluated by thermogravimetric analysis (TGA). A Linseis Model TG-PT1000 thermogravimetric analyzer was used (Selb, Germany). The experiments were performed under dynamic mode from 30 °C to 700 °C at 20 °C/min and under a nitrogen atmosphere (flow rate of 10 mL/min). The weight of all samples ranged between 16 mg and 20 mg. The onset degradation temperatures (T_0_) were determined at 5% mass loss, whereas temperatures at the maximum degradation rate (T_max_) were calculated from the first derivative of the TGA curves (DTG).

##### Differential Scanning Calorimetry

Dynamic differential scanning calorimetry (DSC) experiments were performed with a Mettler-Toledo 821 DSC (Schwerzenbach, Switzerland). The average sample weight was between 8 and 10 mg. Samples of each material were subjected to different thermal cycles: PLA was assayed with a first heating scan from 25 °C to 250 °C, followed by a cooling scan from 250 °C to 25 °C, and then a second heating scan up to 350 °C. Meanwhile, PETG+CF and CPE were subjected to the following thermal cycle: a first heating scan from 25 °C to 350 °C, followed by a cooling scan from 350 °C to 25 °C, and finally with a second heating scan up to 350 °C. The cooling and heating rates of the DSC were 10 °C min^−^^1^, under a N_2_ atmosphere (100 mL min^−^^1^). PETG and CPE are amorphous materials and thus only the glass transition temperature (T_g_) of the 3D printed parts was calculated.

#### 2.3.6. Attenuated Total Reflectance-Fourier Transformed Infrared Spectroscopy

Fourier transformed infrared (FTIR) spectroscopy measurements under attenuated total reflectance (ATR) were used to follow the chemical modifications in a Perkin Elmer Spectrum BX model (Perkin-Elmer, Boston, MA, USA). Spectra were obtained in the range within 4000 cm^−^^1^ and 600 cm^−^^1^.

#### 2.3.7. Statistical Analyses

The data from mechanical, and colorimetric properties were statistically analyzed with OriginPro 8 software from OriginLab (Northampton, MA, USA). The significant differences were assessed at a 95% confidence level according to Tukey’s test using a one-way analysis of variance (ANOVA).

## 3. Results

### 3.1. Mechanical Characterization

**Figure 2:**
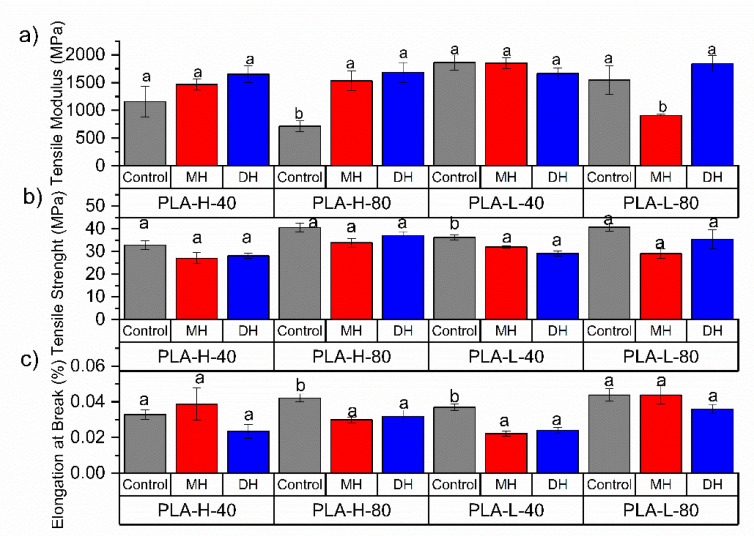
Change in the tensile test properties of the FDM dumbbells due to the DH or MH sterilization process as measured in the PLA 3D-printed samples: (**a**) Tensile Modulus, (**b**) Tensile Strength and (**c**) elongation at break. Grey bar: control sample; blue bar: dry heat sterilization; and red bar: moist heat sterilization. ^a,b^ Different letters show statistically significant differences between formulations (*p* < 0.05).

**Figure 3:**
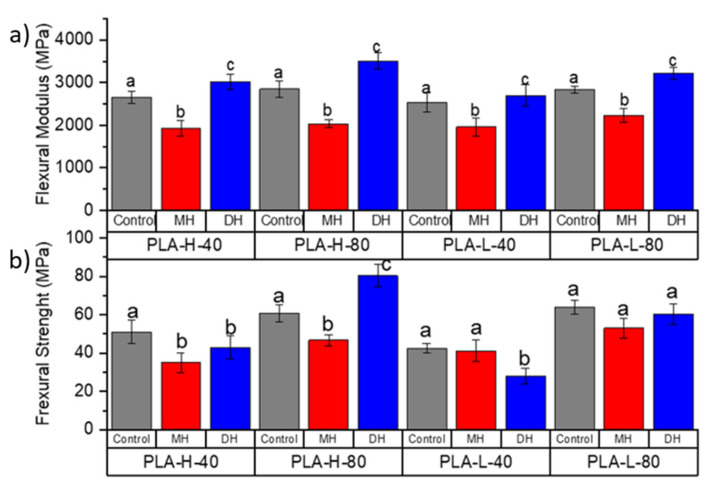
Change in the flexural modulus (**a**) and flexural strength (**b**) of the FDM dumbbells due to the DH or MH sterilization process as measured in the PLA 3D-printed samples. Grey bar: control sample; blue bar: dry heat sterilization; and red bar: moist heat sterilization. ^a–c^ Different letters show statistically significant differences between formulations (*p* < 0.05).

**Figure 4:**
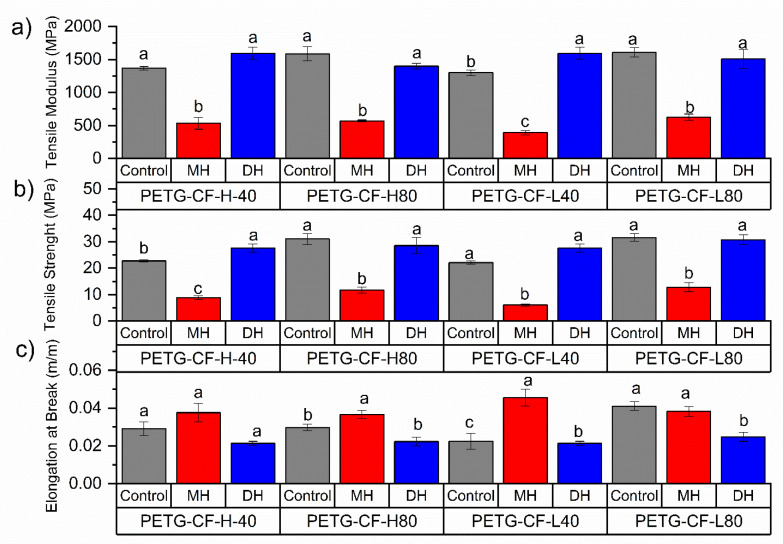
Change in the tensile modulus (**a**), tensile strength (**b**) and elongation at break (**c**) of FDM dumbbells due to the DH or MH sterilization process as measured in the PETG. Grey bar: control sample; blue bar: dry heat sterilization; and red bar: moist heat sterilization. ^a–c^ Different letters show statistically significant differences between formulations (*p* < 0.05).

**Figure 5:**
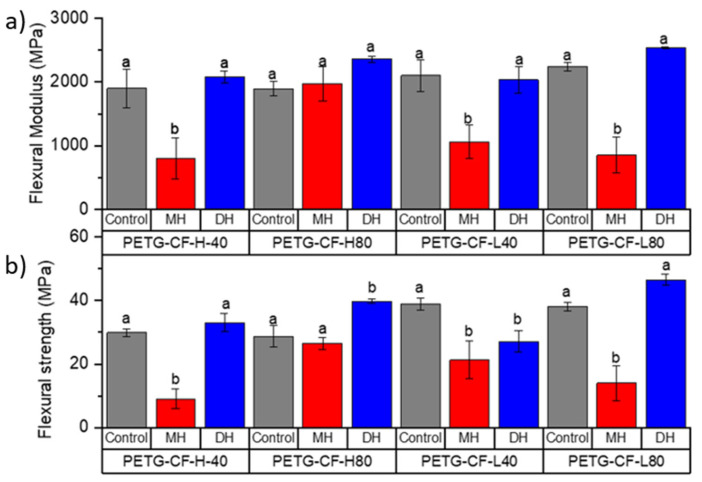
Change in the flexural modulus (**a**) and flexural strength (**b**) of the FDM dumbbells due to the DH or MH sterilization process as measured in the PETG 3D-printed samples. Grey bar: control sample; blue bar: dry heat sterilization; and red bar: moist heat sterilization. ^a,b^ Different letters show statistically significant differences between formulations (*p* < 0.05).

**Figure 6:**
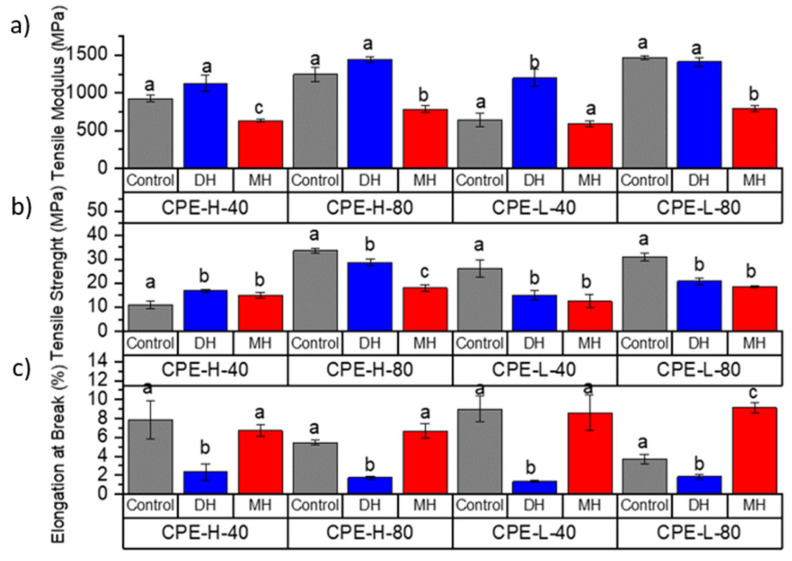
Change in the tensile modulus (**a**), tensile strength (**b**) and elongation at break (**c**) of the FDM dumbbells due to the DH or MH sterilization process as measured in the CPE. Grey bar: control sample; blue bar: dry heat sterilization; and red bar: moist heat sterilization. ^a–c^ Different letters show statistically significant differences between formulations (*p* < 0.05).

**Figure 7:**
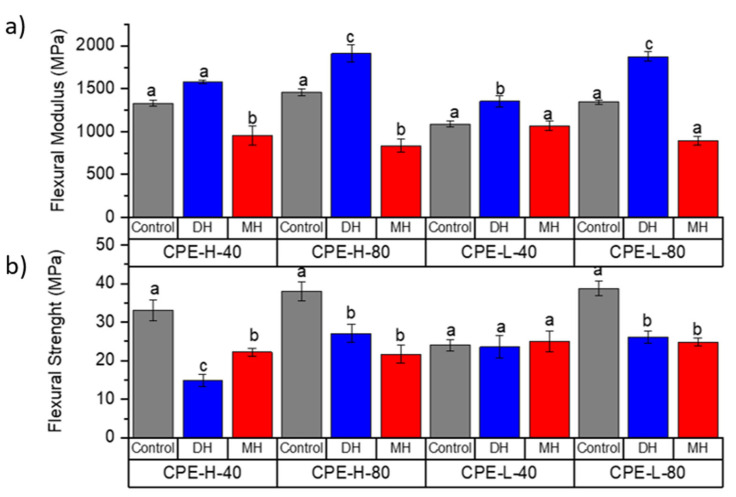
Change in the flexural modulus (**a**) and flexural strength (**b**) of the FDM dumbbells due to the DH or MH sterilization process as measured in the CPE. Grey bar: control sample; blue bar: dry heat sterilization; and red bar: moist heat sterilization. ^a–c^ Different letters show statistically significant differences between formulations (*p* < 0.05).

**Figure 8:**
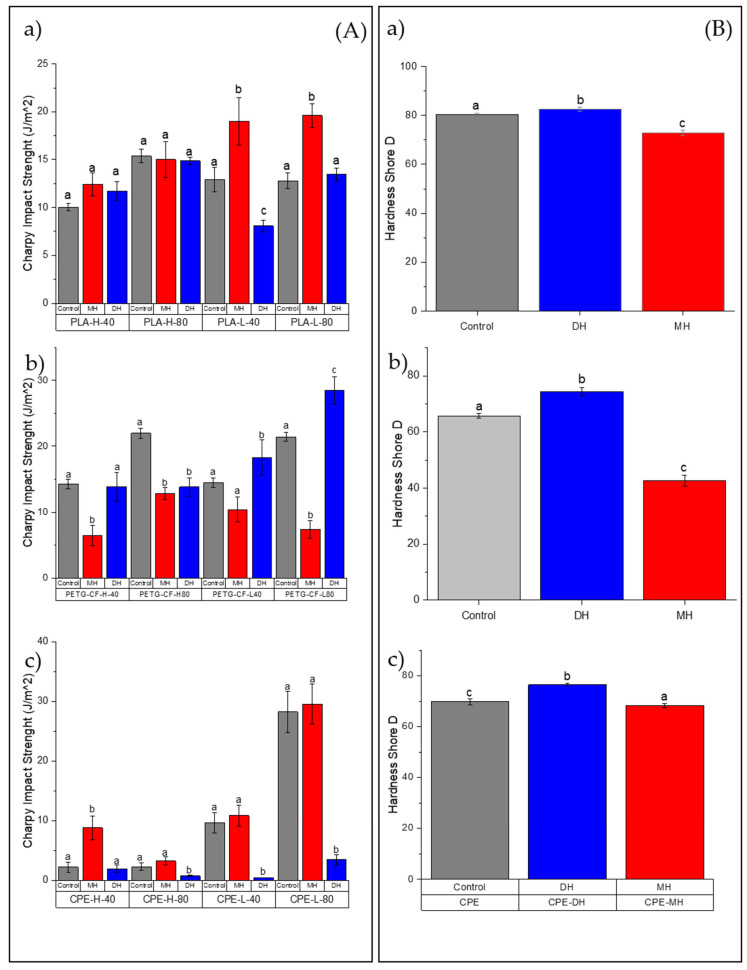
Change in the Charpy impact strength (**A**) and Shore D hardness (**B**) of the FDM dumbbells due to the DH or MH sterilization process as measured in the (a) PLA, (b) PETG-CF and (c) CPE-L. Grey bar: control sample; blue bar: dry heat sterilization; and red bar: moist heat sterilization. ^a–c^ Different letters show statistically significant differences between formulations (*p* < 0.05).

The tensile test and flexural mechanical properties of PLA (Figure 2 and Figure 3), PETG (Figure 4 and Figure 5) and CPE (Figure 6 and Figure 7) were studied. The Charpy impact strength (Figure 8, panel A) and the hardness were also measured (Figure 8, panel B).

The effects of moist heat (MH) and dry heat (DH) sterilization processes on the PLA tensile test properties are shown in Figure 2a–c. The tensile modulus (TM) significantly decreased (Figure 2a, *p* < 0.05) while the EB increased due to the 3D-printing process, which are in good agreement with the decrease in the T_g_ (Table 2). The PLA filament for the FDM process had a tensile strength (TS) of 50.8 MPa (Figure 2b) and an elongation at break (EB) equal to 2.9% (Figure 2c).

Regarding the sterilization treatments, after the moist heat (MH) treatment, the TM was mainly maintained in all samples with the exception of PLA-L-80-MH, in which a reduction in the TM was clearly observed (Figure 2a). Meanwhile, the TS (Figure 2b) and EB (Figure 2c) were mainly maintained after the moist heat treatment in all samples. Sterilized specimens with MH showed smaller flexural modulus (FM, Figure 3a) with a significant reduction (*p* < 0.05) of about 30% (for instance the PLA filament has a FM: 3150 MPa and PLA-H80-MH: 2030.6 MPa). The flexural strength (FS) was only reduced in formulations with honeycomb patterns, while those with rectilinear fill patterns mainly maintained their FS values (Figure 3b). The moist heat sterilization process reduced the bending resistance value of printed PLA parts (51.2 MPa) up to 31% for PLA-H-40-MH (35.1 MPa). Samples decreased in hardness (*p* < 0.05), where on average, the decrease in hardness was 15%.

In general, after the dry heat (DH) sterilization process, TM values were maintained or even increased (PLA-H-80-DH) (Figure 2a), and the TS values were also mainly maintained with the exception of PLA-L-40-DH in which TS was reduced (Figure 2b) with a consequent reduction in the EB where the decrease in EB resulted was up to 35.1% (Figure 2c). However, it should be mentioned that none of the printed specimens reached the manufacturer-defined TM (2.2 ± 0.1 GPa). In all cases, the FM significantly increased (*p* < 0.05), even for infills with 80%, over the module reported by the manufacturer of up to 3386 MPa for the PLA-H-80-DH (Figure 3a). An increase of up to 21.5% in the TM was observed with this process for PLA-L-40. In general, the FM was larger than the unsterilized printed specimens (*p* < 0.05) (for instance, for the PLA-L-40-DH, FM increased up to 21.54% in relation to the PLA-L-40). Meanwhile, a decrease in FS was observed for all DH sterilized PLA samples, except for PLA-H-80-DH, in which a clear increase in the FS was observed (Figure 3b). The maximum reduction in FS is given in the PLA-L-40 specimen with a 34.1% reduction. While the formulations with honeycomb patterns mainly maintained their impact strength values, a significant reduction (*p* < 0.05) in the impact energy absorption was observed for the PLA-L-40-DH sample (Figure 8(Aa)). A slight but significant increment (*p* < 0.05) of their hardness (on average 8%, Figure 8(Ba)) was found and this result is in good agreement with the already commented increase in the TS (Figure 2b) and FS (Figure 3b) values.

The effects of moist heat (MH) and dry heat (DH) sterilization processes on the PETG-CF tensile test properties are shown in Figure 4b. Both the TM (Figure 4a) and TS (Figure 2b) values significantly (*p* < 0.05) decreased as a consequence of the moisture heat (MH) sterilization process, with a consequent principally increment (*p* < 0.05) in the elongation at break (Figure 4c). In fact, in all PETG-CF 3D-printed specimens treated with moist heat, TS drastically decreased by up to 40% as was the case in PETG-L-80-MH (*p* < 0.05). Flexural modulus (FM, Figure 5a) and flexural strength (FS, Figure 5b) decreased due to the moist heat treatment with respect to the 3D-printed PETG-CF sample (*p* < 0.05) with the exception of the PETG-CF-H-80-MH sample, which mainly maintained both values. PETG-CF 3D-printed samples also showed a significant decrease in the impact strength (Figure 8(Ab)) and a significant increase (*p* < 0.05) in the Shore D hardness (Figure 8Bb)).

The dry heat sterilization process did not greatly affect the TM (Figure 4a) and TS (Figure 4b) of the PETG-based materials (*p* > 0.05). Only in the case of the PETG-H-40-DH did the TM result in being greater than that of the control sample (*p* < 0.05). Meanwhile, the EB values shown in Figure 4c were maintained for specimens with infill with the honeycomb type (*p* > 0.05) and slightly reduced for specimens with a rectilinear infill type (*p* > 0.05).

In general, the FM (Figure 5a) and FS (Figure 5b) did not significantly change (*p* > 0.05). While the impact strength (Figure 8(Ab)) in PETG+CF-L-40-DH was maintained due to the DH sterilization process, a decrease in the impact strength in PETG+CF-L-80-DH was observed, and it was significantly increased in the PETG+CF-L-40-DH and PETG+CF-L-80-DH samples (*p* < 0.05). Meanwhile, in contrast to the effect produced by the MH sterilization process, the DH sterilization process produced an increase in the Shore D hardness on an average of 10% (*p* < 0.05) (Figure 8(Bb)).

The effects of moist heat (MH) and dry heat (DH) sterilization processes on CPE tensile test properties are shown in Figure 6. Both the TM (Figure 6a) and the TS (Figure 6b) values were mainly reduced due to the moist heat (MH) sterilization process (*p* < 0.05). In contrast, in CPE-H-40-MH, the TS value increased, and in the rest of the samples, the TS value decreased, mainly showing a maintenance of the elongation at break (Figure 6c), with the exception of the CPE-L-80-MH sample, in which the EB considerably increased (*p* < 0.05). Regarding the flexural modulus (FM, Figure 7a), there was a tendency to reduce this value due to the moisture. In the case of the flexural strength (FS, Figure 7b), a decrease was observed mainly due to the moist heat treatment with respect to the 3D-printed CPE control sample (*p* < 0.05), with the exception of the CPE-L-40-MH sample, which mainly maintained its FS value (*p* > 0.05). Regarding the impact strength (Figure 8(Ac)), there was a tendency to increase due to the moist heat sterilization process, and, in agreement with the other polymeric matrices (PLA and PETG+CF), the moist heat sterilization process produced a significant decrease (*p* < 0.05) in the Shore D hardness (Figure 8Bc)), which was less pronounced.

While the TM (Figure 6a) values were mainly maintained or slightly increased due to the dry heat (DH) sterilization process, the TS (Figure 6b) values significantly (*p* < 0.05) changed. In CPE-H-40-MH, the TS value increased and in the rest of the samples, the TS value decreased, showing a clear reduction (*p* < 0.05) in the elongation at break (Figure 6). Regarding the flexural modulus (FM, Figure 7a), an increase in this property was observed due to the dry heat treatment. Meanwhile, the flexural strength (FS, Figure 7b) showed a more marked decrease (*p* < 0.05), with the exception of the CPE-L-40-MH sample, which mainly maintained the FS value (*p* > 0.05), as occurred in the moist heat treatment. The dry heat produced a reduction in the impact strength, and, in agreement with the other polymeric matrices (PLA and PETG+CF), it produced a significant increase (*p* < 0.05) in the Shore D hardness (Figure 8(Bc)).

Figures S1–S3 represent the tensile test properties as well as the flexural properties of each material studied here. Since there were no significant differences between the rectilinear and honeycomb patterns from the mechanical results, the following characterization was performed with rectilinear fill patterns with 80% fill, since this process is faster and the amount of material is similar.

### 3.2. Microstructural Analysis

**Figure 9:**
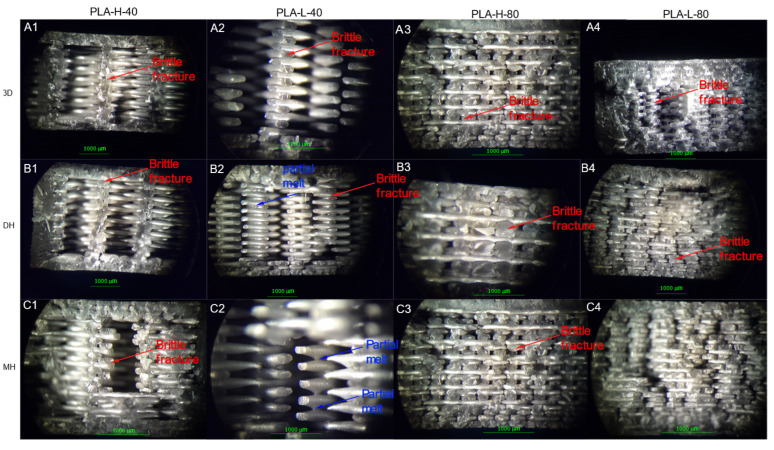
Optical microscope images of the fracture surface of 3the D printed PLA-based samples after the Charpy test. Control samples (without sterilization process treatment): (**A1**–**A4**) PLA-H-40, PLA-L-40 PLA-H-80 and PLA-L-80; samples after the Dry Heat (DH) sterilization process: (**B1**–**B4**) PLA-H-40, PLA-L-40, PLA-H-80 and PLA-L-80; as well as samples after the Moist Heat (MH) sterilization process: (**C1**–**C4**) PLA-H-40, PLA-L-40, PLA-H-80 and PLA-L-80.

**Figure 10:**
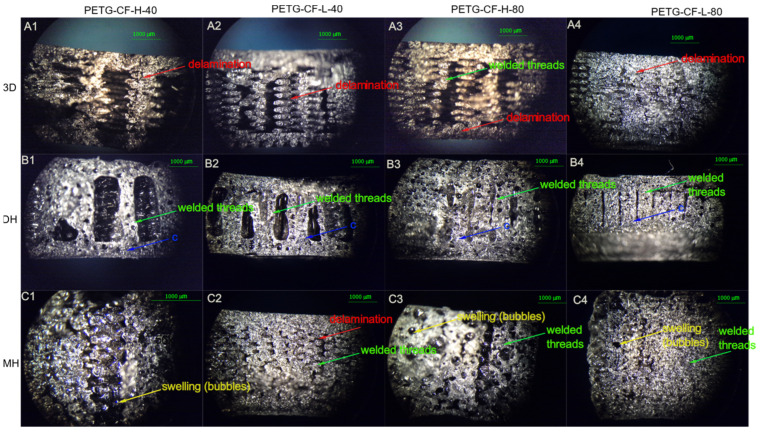
Optical microscope images of the fracture surface of the 3D printed PETG-CF-based samples after Charpy test. Control samples (without sterilization process treatment): (**A1**–**A4**) PETG-H-40, PETG-L-40, PETG-H-80 and PETG -L-80; samples after the Dry Heat (DH) sterilization process: (**B1**–**B4**) PETG-H-40, PETG-L-40, PETG-H-80 and PETG-L-80; as well as samples after the Moist Heat (MH) sterilization process: (**C1**–**C4**) PETG-H-40, PETG-L-40, PETG-H-80 and PETG-L-80.

**Figure 11:**
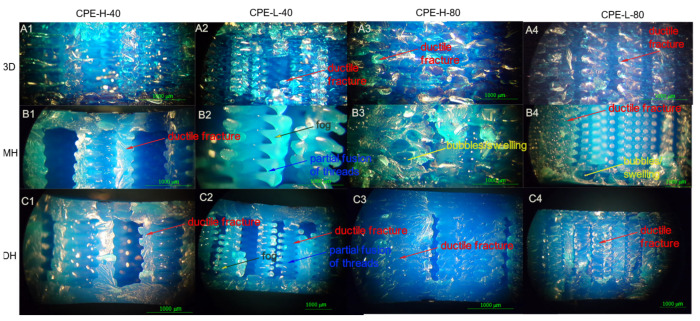
Optical microscope images of the fracture surface of the 3D printed CPE-based samples after the Charpy test. Control samples (without sterilization process treatment): (**A1**) CPE-H-40, (**A2**) CPE-L-40 and (**A3**) CPE-H-80, (**A4**) CPE-L-80; samples after the Dry Heat (DH) sterilization process: (**B1**) CPE-H-40, (**B2**) CPE-L-40, (**B3**) CPE-H-80 and (**B4**) CPE-L-80; as well as samples after the Moist Heat (MH) sterilization process: (**C1**) CPE-H-40, (**C2**) CPE-L-40, (**C3**) CPE-H-80 and (**C4**) CPE-L-80.

The fracture surfaces of the control samples as well as those of the sterilized samples assayed from the Charpy impact test were observed by stereomicroscope and are shown in Figure 9 for the PLA-based samples, Figure 10 for PETG+CF, and in Figure 11 for CPE-based samples. Optical microscopy allows for the immediately inspection of the materials and optical microscopes are frequently available in hospitals, which can allow for the observation of the failure of the 3D-printed parts if the device breaks during surgery. In the case of the PLA-based samples, there was a fragile fracture and no delamination between the printing layers. However, there was a partial fusion of filaments for specimens sterilized by moist heat (Figure 9).

From the visual appearance, the PETG+CF sterilized specimens by moist heat suffered visible deformation due to the presence of bubbles generated by moist heat. Regarding the microstructural analysis, from Figure 10, it can be seen that PETG+CF also showed a fragile fracture and there was no delamination between the printing layers for all PETG+CF-based samples. Partial fusion of filaments for specimens sterilized by moist heat could be observed.

According to Figure 11, CPE also showed a ductile fracture and there was no delamination between the printing layers. There was a partial fusion of filaments for specimens sterilized by moist heat, and some fusion between filaments was also shown after being sterilized by dry heat. Additionally, samples showed color changes as well as in the transparency of the samples after being sterilized with both sterilization processes.

### 3.3. Thermomechanical Analysis

**Figure 12:**
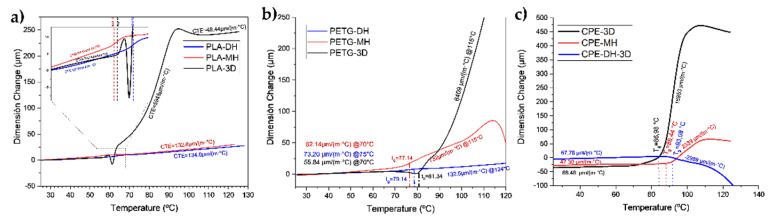
TMA curves of 3D printed (**a**) PLA, (**b**) PETG, and (**c**) CPE-based samples, before and after both sterilization treatments.

The changes in dimensions before and after the sterilization processes were measured by TMA. The TMA curves are shown in Figure 12 and the LCTE before and after T_g_ values are summarized in Table S2.

In Figure 12a, it can be seen that the 3D-printed parts of PLA had a lower coefficient of thermal expansion before T_g_ than the samples sterilized by both moist heat (MH) and dry heat (DH), which indicates that the polymeric chains of PLA-3D have less mobility, therefore, after the sterilization process, the pieces would deform to a greater degree when returning to room temperature. After T_g_, the coefficient of thermal expansion increased dramatically up to 100 °C, where a cold crystallization occurred and the coefficient of thermal expansion decreased again. It has been found that for 3D-printing PLA parts, the higher the printing temperature, the lower the coefficient of thermal expansion, which is caused by different cooling rates during the printing process. When the printing temperature is high, the samples have a longer time to crystallize during cooling and the deformation is lower. With a lower printing temperature, the sample does not have enough time to crystallize, and therefore in the TMA results, the LCTE resulted in being higher.

From Figure 12b, it can be observed that the untreated PETG-CF-based 3D-printed parts had a higher LCTE than the samples sterilized by both moist heat (MH) and dry heat (DH). This indicates that the 3D-pieces would deform as a consequence of the sterilization processes when returning to room temperature. After the DH sterilization process, the pieces would deform to a lesser degree.

LCTE of CPE increased dramatically after T_g_ due to the greater mobility of polymer chains with heat (Figure 12c). It was observed that the T_g_ of CPE—after being subjected to the sterilization process—increased, particularly in the case of dry heat (CPE-DH), suggesting that the material is more rigid, as was observed in the already discussed mechanical characterization.

### 3.4. Colorimetric Measurements

**Figure 13:**
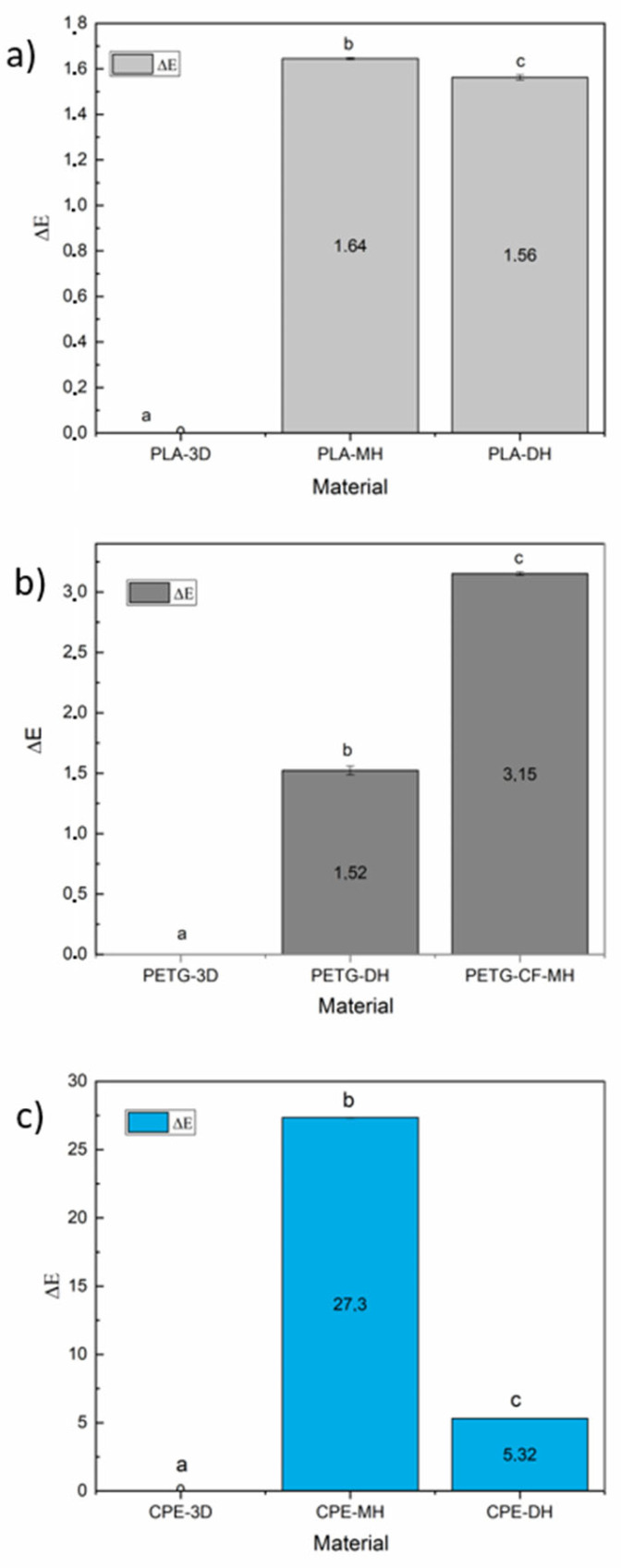
Total color differences before and after the sterilization of the 3D printed samples of (**a**) PLA, (**b**) PETG+CF, and (**c**) CPE-based samples. ^a–c^ Different letters show statistically significant differences between formulations (*p* < 0.05).

The colorimetric properties of the 3D printed samples as well as of sterilized samples by means of moist heat and dry heat sterilization treatments were determined in the CIELab space. The CIELab color coordinates *L* (lightness), a*(+a* = red, −a* = green), and b* (+b* = yellow, −b* = blue) were acquired and the results are in Supplementary Materials (Table S3). Meanwhile, the total color differences are shown in Figure 13.

In the case of the sterilization of PLA samples, it resulted in pieces with an appearance similar to the gray color observed in the control PLA filament. The brightness value (*L*) slightly but significantly decreased (*p* < 0.05) due to both sterilization heat processes (Table S3). Meanwhile, the a* coordinate showed a tendency to green without significant differences between the sterilized samples, and negative b* values were indicative of a trend to blue, which were slightly but significantly increased to more negative values with both sterilization treatments (*p* < 0.05, Table S3). Moreover, the total color differences were significant (Figure 13a).

In the case of the sterilized PETG+CF samples, it resulted in pieces with an appearance similar to the gray color observed in the control PLA filament. The brightness value (*L*) slightly but significantly decreased (*p* < 0.05) due to both sterilization heat processes (Table S3). Meanwhile, the a* coordinate showed a tendency to green without significant differences within sterilized samples, and negative b* values were indicative of a trend to blue, which were slightly but significantly increased to more negative values with both sterilization treatments (*p* < 0.05, Table S3). Moreover, the total color differences were significant (Figure 13b).

Furthermore, in the PETG+CF sterilized samples, a slight whitening of the black specimens could be visually observed, in which their *L* value significantly dropped from 30.1 to 27.0 and 28.6, for specimens sterilized by moist heat and dry heat, respectively (Table S3). The changes in a* and b* coordinates were not significant.

The printed CPE-based 3D-printed parts had a visually observed blue translucent color. The sterilized samples by moist heat had their clarity increased as shown by the significant increase in *L* value, while they practically did not change due to the dry heat sterilization process (Table S3). The a* coordinate showed a tendency to green without significant differences in the dry heat sterilized sample, and with a big tendency toward more greening in the sample with moist heat treatment. The negative b* values, indicative of a trend to blue color, shifted toward more negative values with both sterilization treatments (Table S3). Finally, the total color differences were significant (Figure 13c).

### 3.5. Thermal Characterization

Table 2 summarizes the thermal parameters obtained from DSC and TGA and the obtained thermograms are available in the Supplementary Materials (Figure S4).

PLA mainly crystallized during the 3D printing process and only showed a shoulder during the DSC heating in the region of the cold crystallization temperature (see Figure S4I). The 3D printing process produced a decrease in the T_g_ value with respect to the filament previously to being processed. Similarly, the completed sterilization treatments crystallized the material as the cold crystallization temperature could not be determined in PLA-MH and in PLA-DH. Although there were no significant changes in the melting temperatures, the PLA-DH sample showed two melting peaks (see Figure S4I). The T_g_ value was mainly maintained in the moist heat sterilized sample, while the dry heat produced a higher decrease in the T_g_.

From the DSC curve of the PETG+CF samples (Figure S4II), it can be seen that PETG+CF is an amorphous polymer. For PETG+CF samples, a clear increase in the T_g_ was observed as a consequence of both sterilization treatments, even higher than that of the control PETG+CF (the filament previously to being processed).

The DSC curve of CPE samples (Figure S4III) also showed the typical thermogram of an amorphous polymer. In the case of CPE, the T_g_ value was somewhat reduced due to the 3D printing process, with respect to the filament previously to being processed. Meanwhile, the sterilization treatments produced two different effects. The moist heat treatment produced a decrease in the T_g_ and the dry heat produced an increase in the T_g_, reaching even higher values than that of the control sample.

Regarding the TGA parameters, it was observed that the 3D printing process produced a decrease in the on-set degradation temperature of PLA as well as in the maximum degradation temperature, while both sterilization treatments increased these two values. Comparing the thermal degradation properties of the sterilized 3D printed PLA samples, it can be seen that the on-set degradation temperature (T_5%_) and the degrading temperature (T_95%_) were higher for the sterilized sample with moist heat.

For the PETG+CF samples, the on-set degradation temperature increased due to the 3D printing process and as a consequence of both sterilization treatments, particularly in the case of the moist heat sterilization process. Meanwhile, a different effect took place in the case of the maximum degradation temperature, which shifted to lower values due to the 3D printing process and to higher values due to the sterilization processes.

For the CPE samples, the on-set degradation temperature decreased considerably due to the 3D printing process, while it was increased due to both sterilization treatments, reaching values close to that of the control material. Similarly, the maximum degradation temperature experienced the same tendency.

### 3.6. ATR-FTIR Characterization

**Figure 14:**
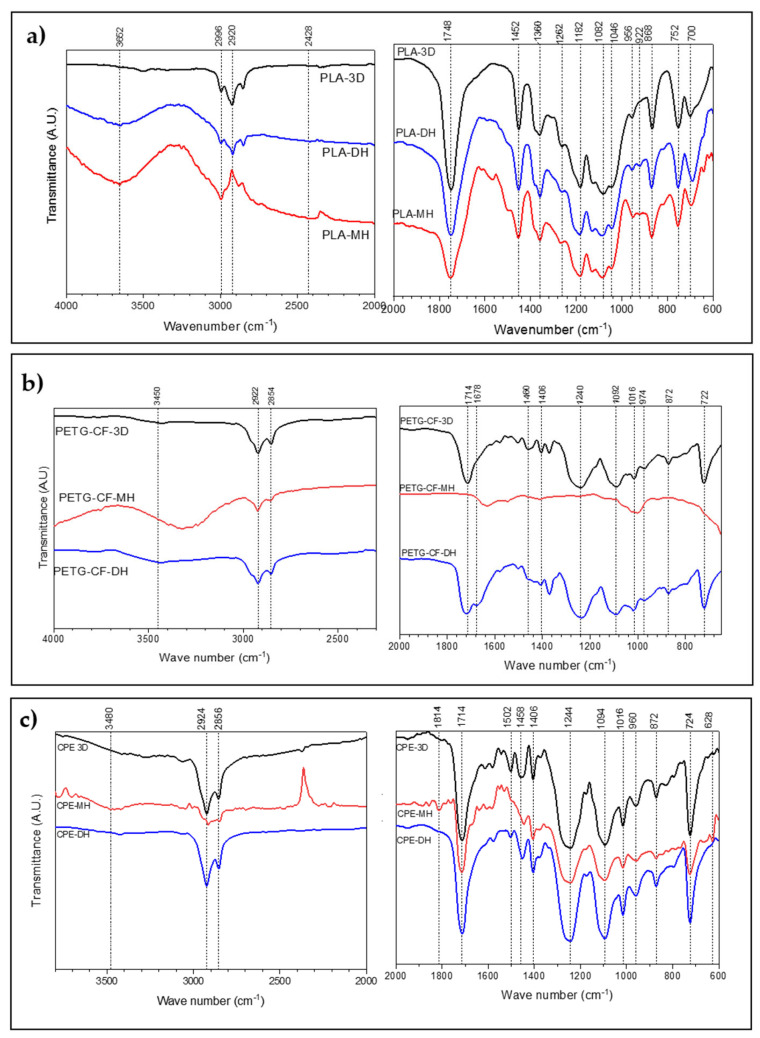
ATR-FTIR analysis before and after the sterilization treatments of 3D printed samples of (**a**) PLA, (**b**) PETG+CF, and (**c**) CPE-based samples.

Figure 14 shows the ATR-FTIR analysis of samples before and after both sterilization treatments. Untreated 3D-printed PLA (Figure 14a) showed a peak at 2996 cm^−^^1^ due to the vibrations of CH_3_ assigned to asymmetric stretching and another one at 2920 cm^−^^1^ for asymmetric stretching. At 1748 cm^−^^1^, carbonyl stretching took place. At 1452 cm^–1^, there was a peak that corresponded to the bending of the methyl group –CH_3_. The symmetric deformation of –CH– occurred at 1360 cm^–1^. There was a stretch of –C–O– at 1182 cm^–1^. The peak at 1082 cm^–1^ was attributed to the symmetric stretching of the –C–O– of the ester groups. The bending of –OH occurred at 1046 cm^−1^. At 868 cm^−1^, there was a peak that corresponded to the stretching of the crystalline phase of PLA, while the amorphous phase was related to the peak at 752 cm^−1^.

Regarding the sterilized samples, an additional peak was shown in the moist heat sterilized PLA sample at 3652 cm^−^^1^. The band at 1452 cm^−1^, which can be attributed to the asymmetric stretching of the methyl –CH_3_ group, shifted slightly at a higher frequency (around 2 cm^−1^). An absorption band appeared at 922 cm^−^^1^. Meanwhile, the intensity of the band at 956 cm^−^^1^ decreased. In the case of the dry heat sterilization process, an absorption band also appeared at 922 cm^−^^1^.

For the PETG+CF samples (Figure 14b), there was a peak at 1714 cm^−^^1^ associated with the stretching of carbonyl ester groups, typical of pure PETG [22]. The two peaks at 1406 cm^−^^1^ and 1240 cm^−^^1^ were attributed to –CH_2_– in its deformation band and to the stretching of ester groups, respectively [28]. The out-of-plane C–H deformation of two carbonyl substitutes in the aromatic ring occurred at 722 cm^−^^1^ [23]. The C–H bending peak of the methylene group was observed at 1460 cm^−^^1^. The peak of elongation of the cyclohexane ring was observed at 974 cm^−^^1^ [29]. Deflection was observed in the 1092 cm^−^^1^ and 1015 cm^−^^1^ bands, which could be caused by the stretching of C–O and the bending of the ether bonds present in the molecule [30].

In the case of the PETG+CF-based 3D-printed samples sterilized by moist heat, relevant peaks could be seen at 3450 cm^−^^1^. The C–H stretching peak of the cyclohexene ring was not found at 974 cm^−^^1^ as well as the peak at 1714 cm^−^^1^, corresponding to the C=O stretching of the ester groups. Meanwhile, the dry heat sterilized samples (PETG-CF-DH) produced a broadening in the carbonyl region, observed between 1750 cm^−^^1^ and 1650 cm^−^^1^, which can be attributed to the formation of terephthalic acid (1678 cm^−^^1^) as end groups [31].

For the CPE 3D-printed samples (Figure 14c), most functional groups of PETG were found since it is a modified PETG. In the 3D-printed CPE moist heat sterilization sample, there was a peak at 3480 cm^−^^1^ corresponding to a stretching of the H bond of the –OH functional group. A decrease in the carbonyl band (1714 cm^−^^1^) was also observed. A peak appeared at 1814 cm^−^^1^, corresponding to stretching of the carbonyl group [32]. At 1640 cm^−^^1^, a peak appeared that is characteristic of the stretching of the carboxylate groups. The 2924 cm^−^^1^ peak shifted to 2916 cm^−^^1^, the 1454 cm^−^^1^ peak shifted to 1446 cm^−^^1^, and the 960 cm^−^^1^ peak shifted to 958 cm^−^^1^. The 724 cm^−^^1^ peak also shifted to 726 cm^−^^1^.

No significant differences were found between the unsterilized 3D-printed CPE and dry heat sterilized CPE.

## 4. Discussion

In the present work, three types of 3D-printing filaments with low processing temperatures—PLA (215 °C), PETG loaded with carbon fibers (PETG+CF, 250 °C), and CPE (275 °C)—were used with the aim to produce sterilizable personalized parts for single-use surgical devices (e.g., screws, surgical guides, etc.). The obtained 3D-printing parts processed through FDM were sterilized by two sterilization processes—moist heat (MH) and dry heat (DH)—and evaluated.

For the PLA-based sample, the mechanical properties revealed that the moist heat (MH) sterilization process produced an increase in the crystallinity of the system due to the absorption of water, as revealed in ATR-FTIR analysis with the appearance of a peak at 3652 cm^−1^, which means that there is residual water due to the hygroscopic nature of PLA [33]. Similarly, the peak at the 1452 cm^−1^ band, attributed to the asymmetric stretching of the methyl –CH_3_ group, shifted slightly to a higher frequency (1454 cm^−1^), which can be related to the changes in the hydrophobic properties of PLA, during hydrolysis. The increase in the crystallinity took place with a consequent increase in the tensile modulus and impact resistance. Although the water can act to plasticize the system, its elongation at break was reduced as was the tensile strength, flexural modulus, flexural strength, and hardness. In fact, the already stated 15% of the decrease in hardness can be attributed to the decrease in PLA crystallinity [16]. Crystallinity in a 3D printed part is not constant as the outer layers cool faster than the inner layers, causing the outer perimeters, in general, to have lower flexural and tensile strength (Figure 2 and Figure 3). The material increased its crystallinity due to slow cooling and because the polymer chain underwent cleavage due to the degradation produced from the moist heat treatment. Morphologically, no changes were observed in the structure and integrity of the printing layers and printed threads, which would indicate that a decrease in some of their mechanical properties is due to the internal change in the structure of the material. After the moist heat sterilization process, the water could not penetrate the crystalline regions of PLA and only penetrated the amorphous phase, leading to the degradation of the amorphous phase in PLA-MH. The degradation of amorphous regions proceeded more rapidly in the bulk of the material than at the surface of the specimens [34]. The degradation produces the crystallization of PLA [6], as demonstrated an absorption band that appeared at 922 cm^−1^, which corresponds to a crystalline phase of PLA [35] as well as the reduction in the intensity of the band at 956 cm^−1^, associated with the decreased amorphous fraction of PLA, which is consistent with the results reported in the literature [36]. The degradation of PLA leads to the cleavage of the polymeric chains, since the external water attacks the ester group (–C=O) and decomposes it into terminal carboxylic acid (–COOH) and alcohol (–OH) residues [37,38]. Thus, the use of this material with the moist heat sterilization process is not recommended.

For the PLA-based sample, the mechanical properties revealed that the dry heat (DH) sterilization process produced embrittlement of the material, as can be observed from the slight increase in the modulus of elasticity, flexural modulus, and Shore-D hardness as well as from the decrease in its flexural strength and elongation at break. From the DSC results, it can be seen that the PLA-DH sample showed two melting peaks, suggesting that there was a formation of different crystals with different thermal stability [39]. The presence of two different crystals populations could be explained by the presence of shorter polymer chains in the dry heat treated material due to the degradation of the polymeric matrix [16] as a consequence of the dry heat treatment. Shorter polymeric chains possess higher mobility, which allows them to rearrange into less-ordered crystalline structures during the heat sterilization treatment and then melt at higher temperatures during DSC heating. Moreover, PLA-DH showed a higher reduction in the T_g_ value, confirming the presence of shorter polymeric chains that act to plasticize the polymeric matrix. From the TGA, it can be confirmed that the DH sterilized sample (PLA-DH) showed less thermal stability than the sterilized sample with MH (PLA-MH). This lower thermal stability can be related to the higher degradation produced in the PLA-DH sample.

The typical yellowing tendency observed in degraded PLA samples due to the hydrolytic process [40] was not observed here, which is believed to be due to the grey color of the specimens partially masking that effect. Nevertheless, from the above-mentioned results, it can be said that although PLA suffers degradation due to both sterilization treatments, it could be used, particularly after dry heat sterilization treatment (PLA-DH), in medical applications in which dimensional accuracy is not a determining factor and there are no great mechanical demands (e.g., surgical guides). With dry heat sterilization treatment, there is an expansion and contraction of the material, which would cause dimensional inaccuracy. Nevertheless, it should be mentioned that chemically, PLA-DH did not undergo significant changes.

For the PETG-CF-based 3D-printed parts, a very different behavior was observed according to the sterilization treatment used. While the moist heat (MH) sterilization mainly decreased the mechanical properties (tensile modulus and, tensile strength; the flexural modulus and strength as well as the Charpy impact resistance and the Shore-D hardness), the dry heat sterilization process mainly maintained the tensile and flexural mechanical properties of the PETG-CF-DH sterilized materials, showing somewhat of an increment in the Charpy impact strength and the Shore-D hardness.

In the PETG-CF sterilized samples with moist heat (MH), the overall mechanical properties decreased, mainly due to the formation of a spongy or bubbly structure in addition to the absorption of water produced by the water vapor, which gives an appearance of swelling. From the ATR-FTIR analysis, the presence of relevant peaks at 3450 cm^−1^ can be assigned to alcohol formation. Additionally, the C–H stretching peak of the cyclohexene ring was not found at 974 cm^−1^, revealing the cleavage of the cyclohexane ring as a degradation product, since the peak corresponded to carbonyl stretching of the ester groups (at 1714 cm^−1^) was not shown. Moreover, it was also observed that the color of the surface changed to a white milky appearance, suggesting degradation. PETG is an amorphous polymer and thus the transparency changes due to the degradation of the polymeric matrix. The cloudy appearance of a transparent polymeric matrix is caused by light scattering, which may appear after long exposure to moisture. Additionally, there were some color changes, indicating that the material suffered degradation. It should be also mentioned that its dimensional accuracy changed, confirming the degradation of the polymeric matrix due to the moist heat sterilization treatment.

In the case of the dry heat (DH) sterilization process of PETG-CF-based 3D-printed materials, the situation was completely different since the overall mechanical resistance was mainly maintained after the dry heat sterilization treatment and no significant visual changes were observed. However, from the ATR-FTIR analysis, a broadening in the carbonyl region (between 1750 cm^−1^ and 1650 cm^−1^) was observed and attributed to the formation of terephthalic acid (1678 cm^−1^) as end groups [31], suggesting that some degradation of the polymeric matrix occurred during the dry heat sterilization treatment. It is presumed that the changes were not significantly noticed visually due to the black color of the filament.

Therefore, the use of PETG-CF for the moist heat (MH) sterilization process is not recommended since the materials suffer hydrolytic degradation, and consequently, all of its mechanical properties decreased. Nevertheless, PETG-CF could be used with dry heat (DH) sterilization processes, if their temperature does not exceed 140 °C, since its tensile mechanical properties are not drastically affected and it dimensional accuracy is not required.

For CPE 3D-printed samples sterilized with the moist heat (MH) sterilization process, although the material maintained its ductility, the tensile modulus, tensile strength, flexural modulus, flexural strength, impact strength, and Shore-D hardness were reduced, indicating a significant loss in the overall mechanical performance. In fact, the decrease in the carbonyl band (1714 cm^−1^), observed from the ATR-FTIR analysis, indicates the degradation of the ester group. Additionally, the appearance of a peak at 1640 cm^−1^, characteristic of the stretching of the carboxylate groups, indicates that some carboxylic acid salt was formed as end groups in the samples exposed to the moist heat sterilization process [41]. The shifts of several peaks (2916 cm^−1^, 1446 cm^−1^ and 958 cm^−1^) would indicate a decrease in the force of the link. The bubbly structure was also observed in the CPE-MH sample as well as the loss of translucency, but was less evident than in the case of PETG-CF-MH.

On the other hand, CPE-DH became embrittled due to its decreasing tensile strength and elongation at break after the dry heat (DH) sterilization treatment. Dimensionally, it will not maintain the required accuracy, since after the glass transition temperature, its linear coefficient of thermal expansion (LCTE) is large. The drastic increase in LCTE after T_g_ implies that the material will undergo considerable deformation when the T_g_ is exceeded with the sterilization processes. The TMA showed that the material had cross-linked due to DH and was more rigid due to the annealing process, which is in accordance with the results of the mechanical tests. Nevertheless, no significant chemical changes were observed between unsterilized 3D-printed CPE and dry heat sterilized CPE samples in the ATR-FTIR analysis and the thermal stability did not decrease. Thus, it can be concluded that no significant degradation took place during the dry heat (DH) sterilization process of CPE-DH.

## 5. Conclusions

It can be indicated that the materials of low processing temperature used here—PLA (220 °C), PETG loaded with carbon fibers (260 °C), and CPE (275 °C)—can be successfully processed by easy, low cost, and accessible processing technology such as FDM into 3D-printed parts intended for single-use surgical applications. To preserve patient safety and guarantee the possibility of using such 3D-printed parts in personalized surgical applications, the obtained 3D-printed materials were sterilized with moist heat (MH) and dry heat (DH) sterilization processes. It was observed that moist heat produced higher degradation of the polymeric matrices than dry heat due to the combination of hydrolytic and thermal degradation of the polymeric matrix. While PETG-CF 3D-printed materials sterilized with a dry heat (DH) sterilization process can be used in medical applications if the temperature does not exceed 140 °C, since its tensile mechanical properties are not drastically affected and its dimensional accuracy is not required, CPE-DH dimensionally will not maintain the required accuracy, since after the glass transition temperature, its coefficient of thermal expansion was large. Although PLA showed signs of degradation after dry heat sterilization treatment (PLA-DH) and there was an expansion and contraction of the material, which can lead to some dimensional inaccuracy, PLA-DH did not undergo significant chemical changes. Thus, PLA-based 3D-printed parts sterilized with a dry heat process can be used in medical applications in which dimensional accuracy is not a key factor and when there are no great mechanical demands (e.g., surgical guides). In summary, FDM offers a good perspective for the development of biobased, easily-produced, and low-cost PLA-based 3D-printing parts for dry heat sterilization to be used as single-use surgical devices.

## Figures and Tables

**Table 1:** Designation of each printed sample in this study, the sterilization process used, as well as the type and the Infill percentage used.

Sample	Infill Percentage	Type of Filling	Sterilization Process
PLA-L-80	80%	Rectilinear-L	-
PLA-L-80-DM	80%	Rectilinear-L	DM
PLA-L-80-MH	80%	Rectilinear-L	MH
PLA-L-40	40%	Rectilinear-L	-
PLA-L-40-DM	40%	Rectilinear-L	DM
PLA-L-40-MH	40%	Rectilinear-L	MH
PLA-H-80	80%	Honeycomb-H	-
PLA-H-80-DM	80%	Honeycomb-H	DM
PLA-H-80-MH	80%	Honeycomb-H	MH
PLA-H-40	40%	Honeycomb-H	-
PLA-H-40-DM	40%	Honeycomb-H	DM
PLA-H-40-MH	40%	Honeycomb-H	MH
PETG+CF-L-80	80%	Rectilinear-L	-
PETG+CF-L-80-DM	80%	Rectilinear-L	DM
PETG+CF-L-80-MH	80%	Rectilinear-L	MH
PETG+CF-L-40	40%	Rectilinear-L	-
PETG+CF-L-40-DM	40%	Rectilinear-L	DM
PETG+CF-L-40-MH	40%	Rectilinear-L	MH
CPE-H-80	80%	Honeycomb-H	-
CPE-H-80-DM	80%	Honeycomb-H	DM
CPE-H-80-MH	80%	Honeycomb-H	MH
CPE-H-40	40%	Honeycomb-H	-
CPE-H-40-DM	40%	Honeycomb-H	DM
CPE-H-40-MH	40%	Honeycomb-H	MH

**Table 2:** DSC and TGA parameters of each printed sample.

Sample	T_g_ (°C)	T_cc_ (°C)	T_m_ (°C)	T_5%_ (°C)	T_max_ (°C)	T_95%_ (°C)
PLA (control)	64.4	114.2	150.5	348.6	380.6	402.8
PLA 3D printed	57.7	112.0	150.1	330.4	365.0	385.0
PLA-MH	57.6	-	152.3	353.5	383.5	404.5
PLA-DH	54.1	-	151.2	342.5	380.0	392.9
PETG (control)	80.0	-	-	340.8	438.8	>700
PETG 3D printed	76.5	-	-	377.5	433.5	>700
PETG-MH	84.3	-	-	414.0	458.5	>700
PETG-DH	85.0	-	-	383.0	453.0	>700
CPE (control)	83.7	-	-	406.2	439.0	>700
CPE 3D printed	81.1	-	-	396.0	428.5	>700
CPE-MH	76.2	-	-	407.5	443.2	>700
CPE-DH	88.9	-	-	406.5	444.0	>700

## Data Availability

All data is available through e-mail (jmfuentes@uce.edu.ec).

## Supplementary Materials

The following supporting information can be downloaded at: https://www.mdpi.com/article/10.3390/polym14050855/s1, Figure S1: Radar charts of the tensile test and flexural mechanical properties of PLA; Figure S2: Radar charts of the tensile test and flexural mechanical properties of PETG; Figure S3: Radar charts of the tensile test and flexural mechanical properties of CPE; Figure S4: DSC and TGA thermograms of (I) PLA, (II) PETG-CF and (III) CPE control dumbbells as well as samples sterilized with DH an MH. Table S1: Main 3D printing parameters of each filament material, obtained from the Slic3r Prusa Edition software; Table S2: DSC and TGA parameters of each printed sample; Table S3: Colorimetric parameters from CIELab space of each 3D-printed sample before and after the sterilization process
