# Supplementary material for: Effects of Steam Heat and Dry Heat Sterilization Processes on 3D Printed Commercial Polymers Printed by Fused Deposition Modeling
Source: Polymers (Basel). 2022 Feb 22;14(5):855. doi: 10.3390/polym14050855 (PMC8912381; doi:10.3390/polym14050855)
Supplement: Supplementary file 1 [file polymers-14-00855-s001.zip › polymers-1569474-supplementary.pdf]

polymers

Supporting Information

# Effects of steam heat and dry heat sterilization processes on 3D printed commercial polymers printed by fused deposition modeling

**Jorge Mauricio Fuentes [1,2,*], Marina Patricia Arrieta [3,4*], Teodomiro Boronat [1] and Santiago Ferrándiz [1,*]**

1 Instituto de Tecnología de Materiales, Departamento de Ingeniería Mecánica y de Materiales, Universitat Politécnica de València, Plaza Ferrándiz y Carbonell s/n, 03801 Alcoi, Spain; tboronat@dimm.upv.es; sferrand@mcm.upv.es
2 Ingeniería en Diseño Industrial, Facultad de Ingeniería y Ciencias Aplicadas, Universidad Central del Ecuador, 170521, Quito, Ecuador; jmfuentes@uce.edu.ec
3 Departamento. Ingeniería Química Industrial y Medio Ambiente, Universidad Politécnica de Madrid, E.T.S.I. Industriales, 28006 Madrid, Spain; m.arrieta@upm.es
4 Grupo de Investigación: Polímeros, Caracterización y Aplicaciones (POLCA), 28006 Madrid, Spain
*Correspondence: jmfuentes@uce.edu.ec (J.M.F.); m.arrieta@upm.es (M.P.A.); sferrand@mcm.upv.es (S.F.)

**Citation:** Lastname, F.; Lastname, F.; Lastname, F. Title. *Polymers* **2021**, *13*, x. https://doi.org/10.3390/xxxxx

Academic Editor: Firstname Lastname

Received: date
Accepted: date
Published: date

**Publisher's Note:** MDPI stays neutral with regard to jurisdictional claims in published maps and institutional affiliations.

**Copyright:** © 2021 by the authors. Submitted for possible open access publication under the terms and conditions of the Creative Commons Attribution (CC BY) license (https://creativecommons.org/licenses/by/4.0/).

**Table S1.** Main 3D printing parameters of each filament material, obtained from the Slic3r Prusa Edition software.

| Characteristic | Slicer Prusa Edition parameter | | PLA | PET-G+CF | CPE |
|---|---|---|---|---|---|
| Filament | Supplier | | Prusament | Nanovia | Fillamentum |
| | Name | | Prusament Generic PLA | PETG | CPE HG100 HM100 |
| | Color | | Grey | Black | Blue |
| | Density | g/cm$^3$ | 1.24 | 1.27 | 1.25 |
| | First layer temperature | °C | 215 | 240 | 275 |
| | Other layer temperature | °C | 210 | 250 | 275 |
| | First layer bed temperature | °C | 60 | 85 | 90 |
| | Other layer bed temperature | °C | 60 | 90 | 90 |
| | Filament diameter | mm | 1.75 | 1.75 | 1.75 |
| Velocity | Perimeter velocity | mm/s | 45 | 45 | 45 |
| | Infill velocity | mm/s | 80 | 80 | 80 |
| Layers and perimeters | Layer height | mm | 0.15 | 0.15 | 0.15 |
| | First layer height | mm | 0.2 | 0.2 | 0.2 |
| | Vertical shell number | | 2 | 2 | 2 |
| Infill | Infill type | | Honeycomb rectilinear | Honeycomb recti-linear | Honeycomb rectilinear |
| | Infill density | % | 40 & 80 | 40 & 80 | 40 & 80 |
| | Infill angle | ° | 45 | 45 | 45 |

**Table S2.** DSC and TGA parameters of each printed sample

| Sample | $T_g$ (°C) | LCTE1 (before $T_g$) (µm/m °C) | $T_{LCT1}$ (°C) | LCTE2 (after $T_g$) (µm/m °C) | $T_{LCT2}$ (°C) |
|---|---|---|---|---|---|
| PLA 3D printed | 55.2 | 73.2 | 55 | 78.4 | 125 |
| PLA-MH | 56.5 | 87.5 | 60 | 133 | 129 |
| PLA-DH | 62.8 | 97.4 | 55 | 134 | 124 |
| PETG+CF 3D printed | 81.3 | 55.8 | 70 | 6409 | 115 |
| PETG+CF-MH | 77.1 | 82.1 | 70 | 732.0 | 115 |
| PETG+CF-DH | 79.1 | 73.2 | 75 | 132.5 | 124 |
| CPE 3D printed | 87.0 | 68.5 | 80 | 16953 | 129 |
| CPE-MH | 89.4 | 47.3 | 80 | 2339 | 105 |
| CPE-DH | 93.1 | 67.8 | 70 | -2989 | 98 |

**Table S3.** Colorimetric parameters from CIELab space of each 3D-printed sample before and after the sterilization process

| Sample | *L* | *a** | *b** | ΔE |
|---|---|---|---|---|
| PLA 3D printed | 57,60±0,01$^a$ | -0,80±0,01$^a$ | -2.20±0,02$^a$ | 0$^a$ |
| PLA-MH | 56,10±0,01$^b$ | -0,90±0,02$^a$ | -1.70±0,03$^b$ | 1.64$^b$ |
| PLA-DH | 56,00±0,01$^c$ | -0,80±0,03$^a$ | -1.90±0,03$^c$ | 1.56$^c$ |
| PETG+CF 3D printed | 30,10±0,01$^a$ | 0,11±0,04$^a$ | -0,00±0,03$^a$ | 0$^a$ |
| PETG+CF-MH | 27,0±0,02$^b$ | 0,11±0.08$^a$ | -0,15±0,03$^a$ | 3.15$^b$ |
| PETG+CF-DH | 28,60±0,03$^c$ | 0,17±0,07$^a$ | 0,31±0,06$^b$ | 1.52$^c$ |
| CPE 3D printed | 31,50±0,01$^a$ | -5,10±0,03$^a$ | -27,40±0,02$^a$ | 0 |
| CPE-MH | 51,00±0,01$^b$ | -22,70±0,03$^b$ | -34,80±0,02$^a$ | 27.3$^b$ |
| CPE-DH | 34,90±0,01$^c$ | -5,10±0,05$^a$ | -31,50±0,02$^c$ | 5.32$^c$ |

The CIELab color coordinates *L* (lightness), *a** (+*a** = red, −*a** = green), and *b** (+*b** = yellow, −*b** = blue); $^{a–c}$ Different letters show statistically significant differences between formulations ($p < 0.05$).

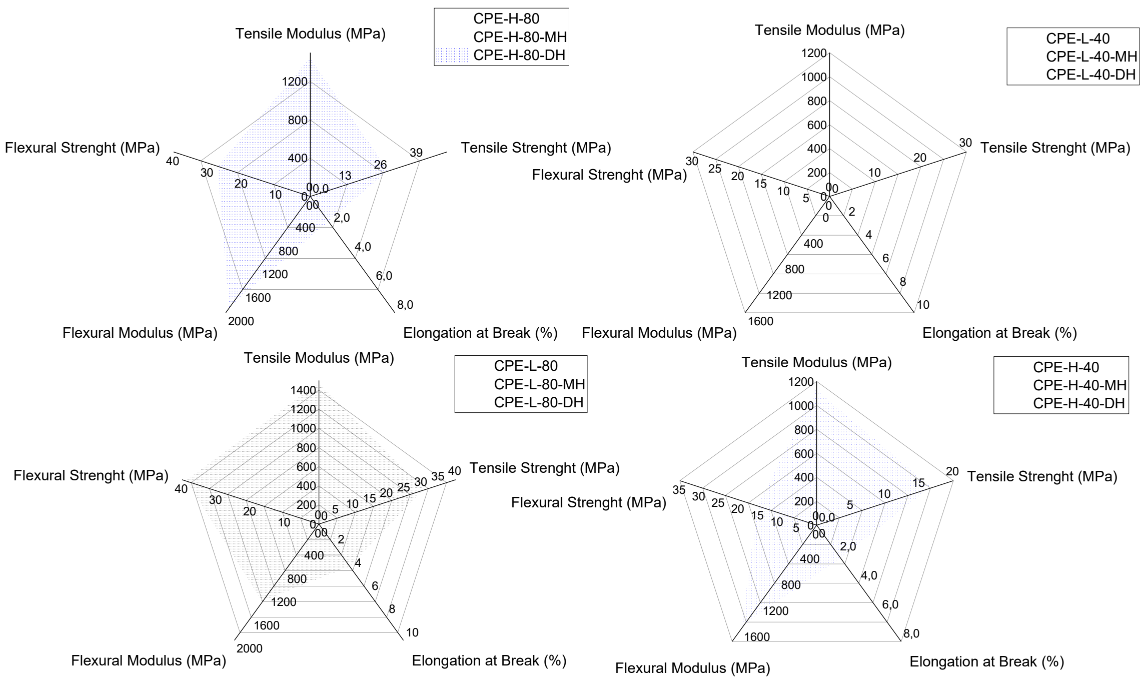


**Figure S1.** Radar charts of the tensile test and flexural mechanical properties of PLA.

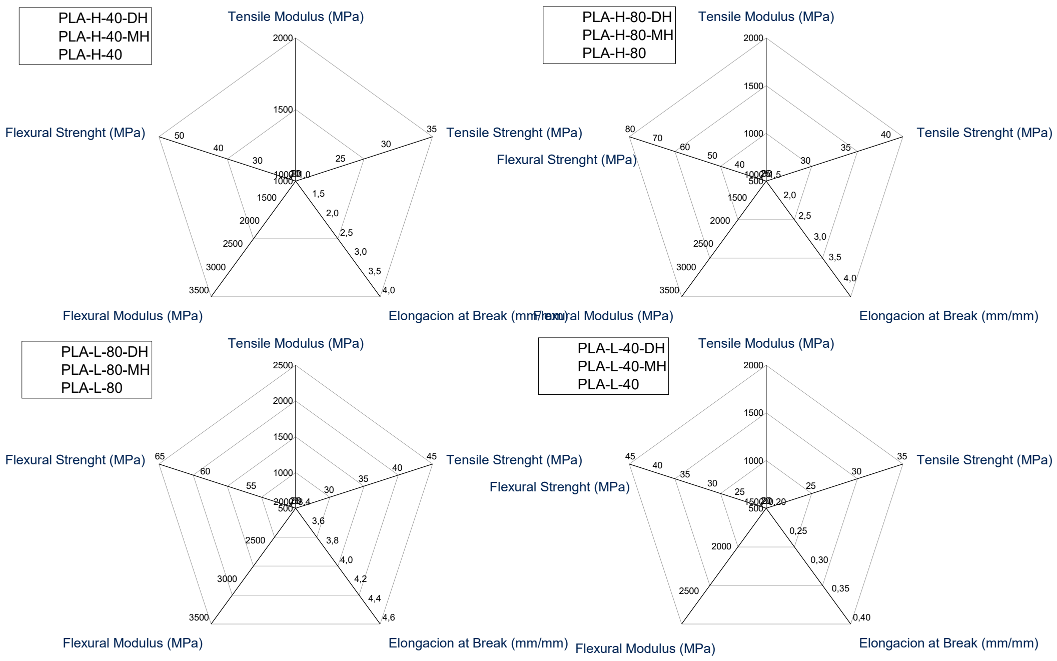


**Figure S2.** Radar charts of the tensile test and flexural mechanical properties of PETG

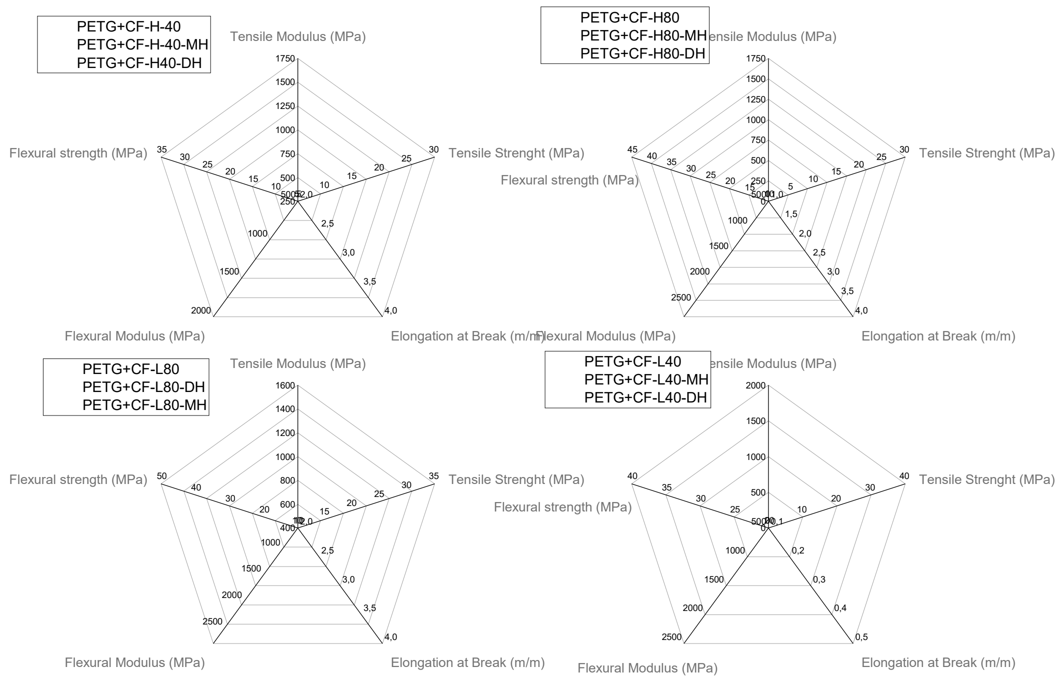


**Figure S3.** Radar charts of the tensile test and flexural mechanical properties of CPE

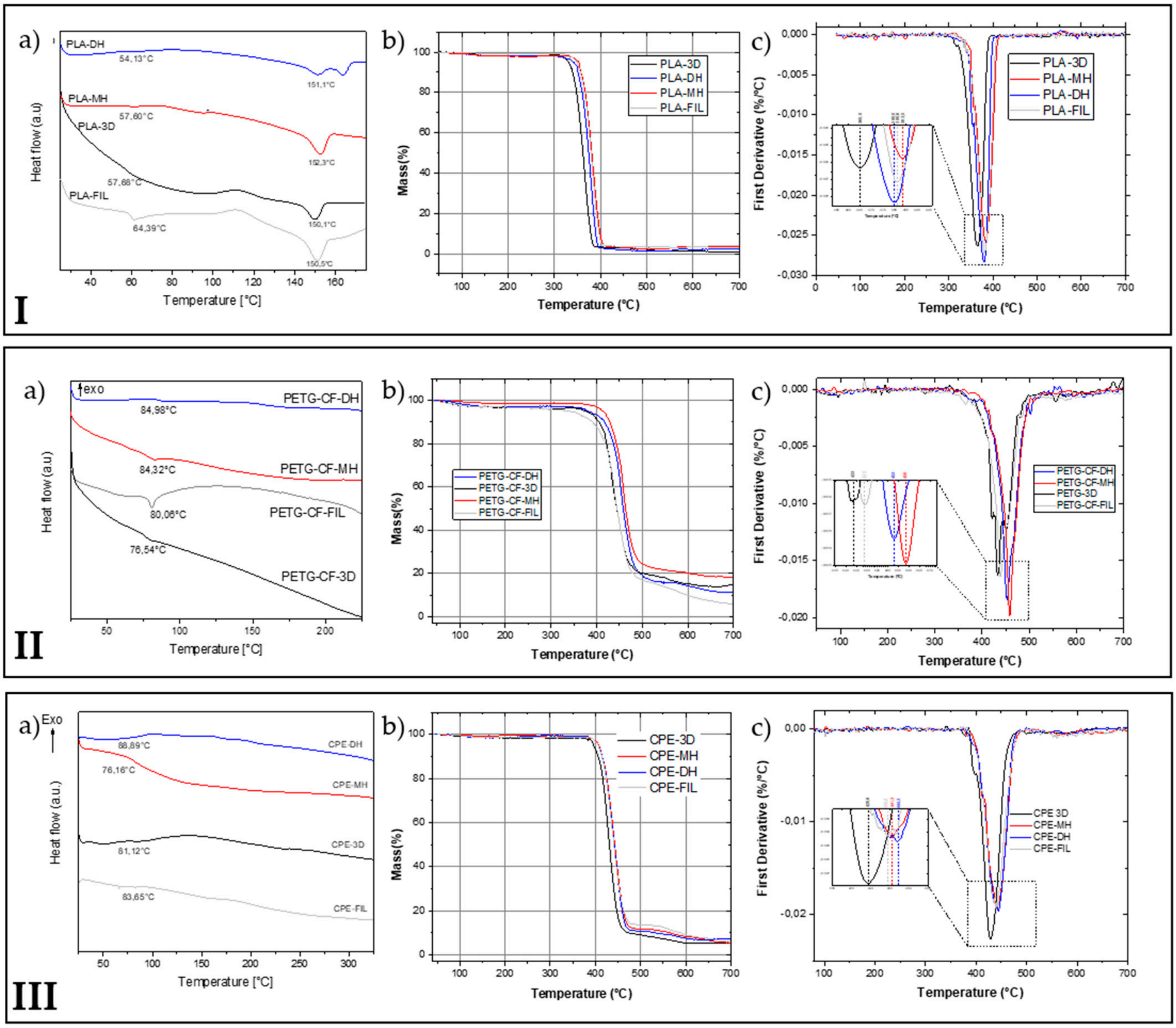


**Figure S4.** DSC and TGA thermograms of I) PLA, II) PETG-CF and III) CPE control dumbbells as well as samples sterilized with DH an MH.
